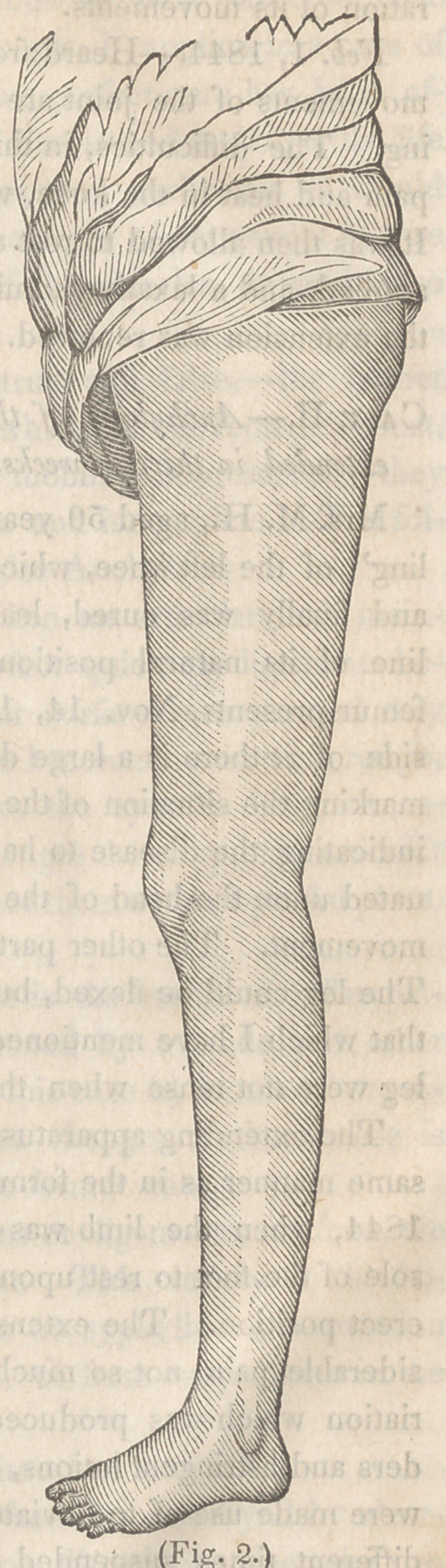# Two Cases of False Anchylosis Cured by Extension, without Division of the Tendons, with Some Remarks upon the Varieties and the Different Modes of Treatment of the Deformity

**Published:** 1844-04

**Authors:** Daniel Brainard


Two cases of False Anchylosis cured by extension, without division of the tendons, with some remarks upon the varieties and the different modes of treatment of the deformity. By Daniel Brainard, M. D.

Case I.—*Anchylosis of the knee, of ten months standing, the result of a wound and suppuration of the joint.—Extended in thirty days.*

T. F. Stevens, of Cook county, Ill., aged about 28 years, of good constitution, consulted me in the early part of August, 1843, for an anchylosis of the left knee. It was the result of an inflammation of the synonial membrane, followed by suppuration, produced by an inscised wound. At this time the suppuration had been stopped ten months, the leg was fixed at a right angle with the thigh, the joint not inflamed, but the fibrous tissues around it were somewhat rigid. On the upper internal part was a cicatrix, more than an inch in length, and the tendons of the flexors of the leg were tense. This I thought a favorable case for gradual extension. The patient being of good constitution, there having been no disease of the bones, and the inflammation having been subdued, were the circumstances which induced the belief that it could be done with safety. As there was no retraction of the muscles, tendons or ligaments, except such as was consequent upon the disease of the joint, I thought it unnecessary to divide these parts, as has sometimes been recommended. The apparatus was applied Aug. 31, 1843, and the leg immediately extended an inch. The adjoining figure (Fig. 1) sufficiently represents its construction, the manner of its application, and the state of the limb when extension had been commenced; *a a* are two concave pieces of brass, fitted to the surfaces of the thigh and leg, and padded before being applied. These are connected together by steel shafts, passing on each side of the knee, and joined opposite to it by hinges. These shafts should be sufficiently strong to resist considerable pressure, and separated so as not to press upon the sides of the knee or the head of the fibula; *b b* &c., are straps for fixing it to the limb, thin cushions of carded cotton should be placed beneath them; *c* is a screw received into the tube *d*, and which contains the female screw. This tube turns upon a pivot at *e*; by turning this forward or back the machine can be varied from a straight line to a right angle; *f* is a buckskin knee-cap for making counter extension, secured to the shafts by six straps, and buckled on each side; *g g* are straps for the same purpose. This instrument, with some slight variations, has been described by Mr. Liston, Dr. Detmold, of New York, Dr. Chase, of Philadelphia, &c., and is frequently sold by the instru-

(Fig. 1.)

(Fig. 2.)

ment makers as the apparatus of one or other of these gentlemen, but was described and figured by Fabricius Hildanus, (J. Cloquet, Dict. de Medicine, Art. Ankylose,) and used with success by Boyer, (Traité des Maladies Chirurgicales, Tome IV. p. 574.)

Extension was made every morning and evening for thirty days, when the limb was perfectly straight, as represented in Fig. 2. The extending apparatus was then removed, a concave splint of tin substituted; the patient returned home, directions being given him in regard to the use of passive motion and frictions for the restoration of its movements.

*Feb.* 1, 1844.—Heard from him,—he walks very well, but the movements of the joint are limited, from the rigidity still remaining. The difficulties, in this case, consisted in the occurrence of pain and heat in the knee, when the extension was carried too far. It was then allowed to rest a day or two; evaporating lotions were applied, and a laxative administered. As the irritation subsided, the extension was renewed.

Case II.—*Anchylosis of the knee of nine years standing.—Leg extended in thirteen weeks.*

Mrs. M. H., aged 50 years, had nine years ago, a "white swelling" of the left knee, which suppurated, continued many months, and finally was cured, leaving the leg flexed to 60° from the line of its natural position when extended. The head of the femur presents, Nov. 14, 1843, an enlargement, and on the outside of it there is a large depressed cicatrix adhering to the bone, marking the situation of the former ulceration of the soft parts and indicating the disease to have been a caries. The patella was situated upon the head of the femur, adherent, but capable of slight movement. The other parts of the joint bore no traces of disease. The leg could be flexed, but not extended to a greater extent than that which I have mentioned. The tendons of the flexors of the leg were not tense when the limb was in a state of repose.

The extending apparatus was applied Nov. 14, 1843, in the same manner as in the former case, and continued until Feb. 14, 1844, when the limb was sufficiently straightened to allow the sole of the foot to rest upon the floor, when the patient was in the erect position. The extension in this case was attended with considerable pain, not so much from inflammation, as from the excoriation which was produced about the cicatrix. Absorbent powders and astringent lotions, with thick cushions under the knee-cap were made use of to obviate this difficulty, but the extension was at different times suspended for several days to allow them to heal. The admirable fortitude and perseverance of the patient, however, were proof against all obstacles, and she cheerfully supported the pain until the desired object was accomplished.

I have thus described two cases of anchylosis and their treatment, in order to bring before the profession the advantages of its adoption in certain varieties of the disease. In order to determine, however, the cases in which it is applicable, and thus be secure against the danger of doing great injury by its use, it is essential to distinguish with accuracy the character of each particular case which presents itself. Thus, for example, if applied in a case of scrofulous disease of the bones before a perfect cure has been effected, it might induce a return of the disease and endanger the life of the patient. If advised in a case of true anchylosis it would but disappoint the patient, and in some cases of permanent flexure of the joints, from spasmodic action of the muscles, it would also be entirely ineffectual.

Anchylosis has been divided into true and false,—the former term being applied to those cases in which all movement is lost, the latter to those in which a partial mobility is preserved; they have also been denominated complete and incomplete. The following are the principal varieties of false Anchylosis:

1st. From want of movement. When an articulation is maintained for a long time in a state of perfect quiet, the ligaments become contracted and rigid, its articular surfaces are pressed very firmly together, the synonial membrane becomes contracted, rough, and firmly adherent by cellular tissue, the synovia becomes serous, diminished in quantity, and at length dried up, the articular cartilegeses are thinned, and ossification of them even may take place. (Dict. de Med. Art Anchylose.)

2d. From effusion of lymph. This is the result of inflammation of the synonial membrane, produced by wounds and other causes. The surfaces are joined in this case by bands of organized false membrane. The subsequent changes, if the disease is left to itself, may be the same as in the former case.

3d. From contraction of the tendons or ligaments, or from the formation of cicatrices about the joint. This variety is the frequent result of rheumatic, scrofulous or syphylitic inflammation of the joints, the formation of eschars, or from suppuration in the cellula tissue.

True Anchylosis may be divided into two kinds:

1st. That in which the articular surfaces are destroyed by ulcerations, and osseous union takes place directly between the two bones. The same result may also take place from the long continuance of false anchylosis.

2d. That in which the bony union takes place without the articulation, from the development of bony tumors, &c. These may arise from fractures, from gouty concretions, &c.

Although we have arranged under the head of true Anchylosis only those cases in which union by callous takes place, it must not be supposed that in the other varieties perceptible movement is always allowed. This is not the case. But as the treatment differs widely in one or the other case we have preferred to classify them in this manner.

It is in the different forms of false Anchylosis that the treatment by gradual extension, or by passive motion, frictions, &c., is most worthy of trial, provided the acute stage be passed and the bones are not diseased. The cases already given may be considered sufficient in regard to extension. As an example of the effect of friction and passive motion, we would cite the following, given by L. Verduc. "A young girl, from 10 to 12 years of age, had the right knee anchylosed, the result of a wound between the condyle of the tibia and the patella. This had been formed seven or eight months, during which the heel was applied to the hip. This disease was regarded as incurable by a physician and three surgeons. Nevertheless Verduc did not despair of restoring her, and undertook its cure. He commenced at first with emollients, and after these, made use of resolvents. These topical applications were continued twice a day with great care during five months. After having fomented the part as warmly as possible for a quarter of an hour with an emollient decoction, he applied the resolvent liquid as warm as she could bear it, and continued to foment it during a considerable time.

But what advanced still more the cure was the bandage, with the splints used for fractures of the leg, the application of which was commenced when the leg was somewhat extended. After having fomented the knee with liquids, he seized the leg and the thigh with his two hands and performed flexion and extension to as great an extent as he was able and the strength of the patient would allow. Afterwards he applied the bandage, preparing a very thin splint, an inch wide and eight or ten inches long, which was placed in a compress of eight folds; he placed the middle of this behind the ham so that one end rested upon the thigh and the other upon the leg. As the leg was still flexed, and there was a wide space between the splint and the ham he put upon the knee another compress, seven or eight double, covered with pasteboard. The bandage was composed of a roller five yards long and two inches wide. Five or six circular turns were made over the compresses, viz., three above and three below the knee, and the roller was then fixed by two or three turns upon the knee itself. It should be observed that in proportion as the anchylosis was softened by the emollient and resolvent remedies, the bandage was tightened. Every day, morning and evening, the flexion and extension were made with violence; in these extended movements the friction between the tibia and the condyles of the femur was heard. All this could not be done without very great pain, which rendered caution necessary. Frequently after having performed these movements it was necessary to leave the patient in a state of repose seven or eight days, and as soon as she was better the flexion and extension were recommenced. By these different means combined, this anchylosis was so perfectly cured that the patient walks since without lameness and without feeling the slightest inconvenience." (Boyer, Op. Cit. Tome IV.) In this case effects which were only due to the mechanical means employed were attributed to the emollients, &c., but it only shows the possibility of effecting by perseverance and great violence what is now accomplished by milder means, such as gradual extension with or without division of the tendons. It is in cases of this kind also that sudden violence sometimes has the effect of restoring the movements. Thus a case has come within my knowledge of a man who had an angular anchylosis of the knee, and who fell down a bank about fifteen feet high, by which the adhesions were ruptured and the person now enjoys the perfect use of the limb. Job à Meek 'ren relates a case which probably depended upon the same cause, and which was cured in a similar manner. "This anchylosis had resisted fomentations and cataplasms; he got a violent fall upon the fore arm, and from that time the movements were rëestablished, and became thenceforth from day to day more extended and easy." (Ap. Cit.)

In cases of disease of the joint attended with flexion, every attempt at extension, or any mechanical violence, must be carefully avoided. This caution is particularly necessary at the present time, when the use of the extending apparatus has been so generally revived. A case has fallen under my notice of a patient with anchylosis of both knees, of several years standing, the legs being flexed to an accute angle with the thighs. The flexon tendons were divided, the apparatus applied, and the limbs nearly straightened, but an inflammation of the joints supervened, which was severe and long continued, rendering the removal of the machines necessary, and terminated by leaving the members in all respects as before the operation. It is probable that the frequent occurrence of such accidents, from improper application of extension, may have contributed to bring this method of treatment to the neglect in which it long slumbered.

The attempt to imitate, by art, those cases in which restoration of motion has been effected by accidental violence have been, as far as I am aware, exceedingly unfortunate. The profession is already aware of the result of two trials by a charletan permitted by Velpeau, in his wards, at the hospital *La Charité.* The following account of the dissection of the joint in the first case, and of the operation in the second, from notes taken at the time, may however add some interesting details. The operation was performed Dec. 29, 1839. The subject was a healthy woman of 25 years of age, with the leg bent to a right angle with the thigh. It was the result of a wound, slight movement was allowed between the tibia and femur, but the patella was fixed to the latter bone. The patient was placed upon the table, the limb placed in the machine, which, by simple turning of a key, immediately brought the knee down to the horizontal piece. This was the work of a minute, but was attended with a cracking sound, from the rupture of tendons, ligaments, &c., which was distinctly audible to all persons within the amphitheatre, and with the most excruciating suffering. The patient while undergoing it, reminding one of a sufferer under instruments of torture. In a week an eschar formed and she died January 19, 1840; but, as she left the hospital, the joint was not examined.

In a case previously operated, and which also terminated fatally, the tibia was found dislocated from the femur, the anterior part of the head of the former bone lying upon the posterior part of the condyles of the latter. Neither the tendons, nerves, or vessels were ruptured. This operation was only intended, as M. Velpan remarked, to apply to true anchylosis.

After these results it is probable that the experiment will not soon be repeated. The only method that has as yet succeeded is the admirable operation of Dr. Barton, which consists in the removal of a wedge from the femur, and which, in the only cases in which it has been repeated, those of Drs. Mütter and Gibson, has been crowned with success.

## Figures and Tables

**Fig. 1. f1:**
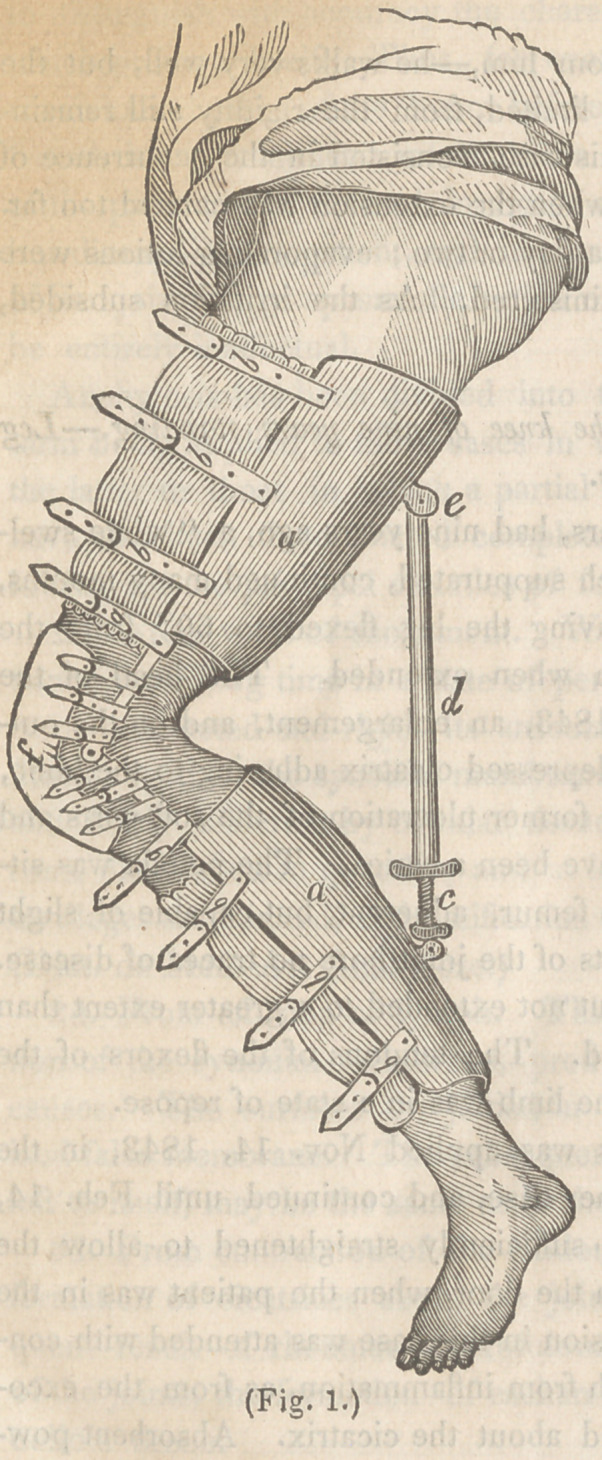


**Fig. 2. f2:**